# Supplementary material for: An Accurate Alternative to Hybrid Functionals for Germanium: DFT+α
Source: J Phys Chem C Nanomater Interfaces. 2026 Jan 7;130(3):1307–11. doi: 10.1021/acs.jpcc.5c05816 (PMC12833989; doi:10.1021/acs.jpcc.5c05816)
Supplement: Supplementary file 1 [file jp5c05816_si_001.pdf]

Supporting Information: An accurate alternative to hybrid functionals for germanium: DFT+α

Abdulgaffar Abdurrazaq,[a,b] Ruggero Lot,[a,c] Antoine Jay,[a] Gabriela Herrero-Saboya,[d,*] Nicolas Richard,[e] Layla Martin-Samos,[d] Anne Hémeryck,[a] Stefano de Gironcoli[b,d]

[a]LAAS-CNRS, Université de Toulouse, CNRS, F-31400 Toulouse, France

[b]SISSA–Scuola Internazionale Superiore di Studi Avanzati, I-34136 Trieste, Italy

[c]AREA Science Park, I-34149 Trieste, Italy

[d]CNR-Istituto Officina dei Materiali (IOM), c/o SISSA, I-34136 Trieste, Italy

[e]CEA, DAM, DIF, F-91297 Arpajon, France

*Corresponding Author: Gabriela Herrero-Saboya
E-mail: herrero@iom.cnr.it

S1 Germanium's band structure

The influence of α in the Kohn-Sham (KS) eigenvalues and cohesive energies is illustrated in Figure S1. We compare DFT+α (PBE+α, α=1.4, black curves) with standard DFT (PBE, red curves) at various lattice constants. In the top panel, we trace the evolution of KS energies from the 4s and 4p atomic orbitals at large lattice constants (a = 15 Å) to the hybridized sp^3 bands. From this figure, it is clear that DFT+α shifts the energy of the 4s-like bands while leaving the 4p-like bands unaffected. At the optimized lattice constant (dashed lines in Figure S1, the direct band gap is closed under standard DFT, whereas DFT+α opens an indirect band gap. This transition is clearly visible in the two zoomed-in panels of the figure.

S2 Related semiconductors

DFT+α addresses a major drawback of commonly used exchange–correlation functionals: the sp mixing in germanium, which leads to a closed band gap. Since sp mixing in diamond and zinc-blende lattices can be a recurring limitation, we show the transferability of the DFT+α approach to related semiconductors. In particular, we focus on α-Sn, GaAs, InAs, GaSb, and InSb.

To asses transferability of the DFT+α framework, Table S1 reports the estimated fundamental band gaps and optimized lattice parameters for the selected semiconductors. The method is applied to other elements by defining $\phi_s(r)$ [Eq. (3) of the manuscript] as the 4s or 5s atomic orbital of the corresponding pseudopotential. For the additional elements present in this benchmark, we use pseudopotentials from the Quantum ESPRESSO PS library[1], which requires a plane-wave basis set with a kinetic energy cutoff of 100 Ry (and 600 Ry for the density cutoff). The Brillouin zone is sampled using a 12^3 Monkhorst–Pack grid.

For this preliminary benchmark, the value of α is kept at 1.4 for elements in the fourth row (Ga, Ge and As). For elements in the fifth row (In, Sn and Sb), α is fixed to 3.0, which makes α-tin a zero-gap semiconductor[2].

From table S1, it is clear that DFT+α improves the description of all considered semiconductors. Al-

TABLE S1: Fundamental band gaps and optimal lattice parameters of semiconductors with diamond and zinc-blende lattices. The DFT+α scheme is compared to the standard PBE functional (DFT) and experimental values [2,3]. The fundamental gaps are measured at low temperatures, while the lattice parameters are determined at room temperature [2]. Only the lattice parameter for Ge is given at low temperatures [3].

Material	Band gap (eV)			Lattice parameter (Å)		
	DFT	DFT + α	Exp. [2]	DFT	DFT + α	Exp. [2,3]
Ge	Metal	0.74	0.74	5.769	5.676	5.652
α-Sn	Metal	0	0	6.644	6.528	6.489
GaAs	0.16	1.06	1.52	5.750	5.686	5.653
GaSb	Metal	0.72	0.82	6.212	6.141	6.096
InAs	Metal	0.22	0.42	6.191	6.118	6.058
InSb	Metal	0.24	0.23	6.633	6.553	6.479

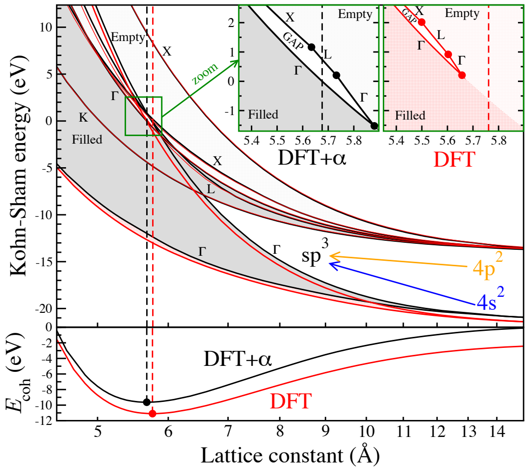

Figure S1. Germanium's band structure and cohesive energy at various lattice constants. Red curves: Pure PBE. Black curves: DFT+α with α = 1.4. Top panel: Kohn-Sham electronic energy obtained from self-consistent field calculations. The energy reference corresponds to the highest occupied band at Γ for the DFT+α lattice with the lowest energy (a_{opt}=5.676 Å). The filled areas correspond to the filled bands, and the dashed area to the empty bands. The high symmetry directions of the Brillouin zone are highlighted by the solid lines (indicated Γ, X, L, K). The inserts are a zoom of the gap region. Bottom panel: cohesive energy of the 2-atom unit cell. The reference cohesive energy is that of non-interacting Ge atoms (unit-cell with a=15 Å), calculated with DFT+α.

though the α values could be further tuned for better accuracy, using one reference value per row provides a reasonable description of *sp* mixing in diamond and zinc-blende lattices. A more detailed benchmark of bulk properties of these semiconductors is beyond the scope of the present work and it will be the subject of a future work.

References

[1] Pseudopotentials for Sn, Ga, As, In and Sb atoms were taken from the *Quantum Espresso PseudoPotential Download Page*: `http://www.quantum-espresso.org/legacy_tables` (files: `Sn.pbe-dn-kjpaw_psl.1.0.0.UPF`, `Ga.pbe-dn-kjpaw_psl.1.0.0.UPF`, `As.pbe-n-kjpaw_psl.1.0.0.UPF`, `In.pbe-dn-kjpaw_psl.1.0.0.UPF`, `Sb.pbe-n-kjpaw_psl.1.0.0.UPF`).

[2] Madelung, O. *Semiconductors: Data Handbook;* Springer: 2004.

[3] Hu, M. Y.; Sinn, H.; Alatas, A.; Sturhahn, W.; Alp, E. E.; Wille, H. C.; Shvyd'ko, Y. V.; Sutter, J. P.; Bandaru, J.; Haller, E. E.; Ozhogin, V. I.; Rodriguez, S.; Colella, R.; Kartheuser, E.; Villeret, M. A. Effect of isotopic composition on the lattice parameter of germanium measured by x-ray backscattering, *Phys. Rev. B* **2003,** *67*, 113306.
